# Imaging of traumatic mandibular fractures in young adults using CT-like MRI: a feasibility study

**DOI:** 10.1007/s00784-022-04736-y

**Published:** 2022-10-08

**Authors:** Georg C. Feuerriegel, Lucas M. Ritschl, Nico Sollmann, Benjamin Palla, Yannik Leonhardt, Lisa Maier, Florian T. Gassert, Dimitrios C. Karampinos, Marcus R. Makowski, Claus Zimmer, Klaus-Dietrich Wolff, Monika Probst, Andreas M. Fichter, Egon Burian

**Affiliations:** 1 Department of Diagnostic and Interventional Radiology, Klinikum Rechts Der Isar, School of Medicine, Technical University of Munich, Munich, Germany; 2 Department of Oral and Maxillofacial Surgery, Klinikum Rechts Der Isar, School of Medicine, Technical University of Munich, Munich, Germany; 3 Department of Diagnostic and Interventional Neuroradiology, Klinikum Rechts Der Isar, School of Medicine, Technical University of Munich, Munich, Germany; 4 TUM-Neuroimaging Center, Klinikum Rechts Der Isar, Technical University of Munich, Munich, Germany; 5 Department of Diagnostic and Interventional Radiology, University Hospital Ulm, Ulm, Germany; 6 Department of Oral and Maxillofacial Surgery, University of Illinois Chicago, Chicago, USA

**Keywords:** Mandibular fracture, CT-like MRI, Traumatic fracture, T1 GRE sequence, Mandible

## Abstract

**Objectives:**

To assess and compare the diagnostic performance of CT-like images based on a three- dimensional (3D) T1-weighted spoiled gradient-echo sequence (3D T1 GRE) with CT in patients with acute traumatic fractures of the mandible.

**Materials and methods:**

Subjects with acute mandibular fractures diagnosed on conventional CT were prospectively recruited and received an additional 3 T MRI with a CT-like 3D T1 GRE sequence. The images were assessed by two radiologists with regard to fracture localization, degree of dislocation, and number of fragments. Bone to soft tissue contrast, diagnostic confidence, artifacts, and overall image quality were rated using a five-point Likert-scale. Agreement of measurements was assessed using an independent *t*-test.

**Results:**

Fourteen subjects and 22 fracture sites were included (26 ± 3.9 years; 4 females, 10 males). All traumatic fractures were accurately detected on CT-like MRI (*n* = 22, κ 1.00 (95% *CI* 1.00–1.00)). There was no statistically significant difference in the assessment of the fracture dislocation (axial mean difference (MD) 0.06 mm, *p* = 0.93, coronal MD, 0.08 mm, *p* = 0.89 and sagittal MD, 0.04 mm, *p* = 0.96). The agreement for the fracture classification as well as the inter- and intra-rater agreement was excellent (range κ 0.92–0.98 (95% *CI* 0.96–0.99)).

**Conclusion:**

Assessment of mandibular fractures was feasible and accurate using CT-like MRI based on a 3D T1 GRE sequence and is comparable to conventional CT.

**Clinical relevance:**

For the assessment of acute mandibular fractures, CT-like MRI might become a useful alternative to CT in order to reduce radiation exposure particularly in young patients.

## Introduction

Mandibular fractures are common fractures of the maxillofacial skeleton, most often occurring in young males between 20 and 30 years of age [1–5]. Depending on the country, time period, and socioeconomic status, the most common causes of mandibular fractures are motor vehicle accidents or direct assaults [6, 7]. Accurate assessment will dictate appropriate surgical treatment and is vital to avoiding complications such as malunion, non-union, risk of infection and osteomyelitis, malocclusion, and neurosensory changes, which all can have significant impact on a patient’s quality of life [1, 8].

Routine diagnostic work-up for mandible fractures includes a thorough clinical examination, in addition to imaging with either two-dimensional (2D) conventional radiographs, computed tomography (CT), or cone beam CT (CBCT) [9]. Due to its high resolution, good bone contrast, wide availability, and low cost, CT is the current reference standard when assessing fractures of the mandible [1]. However, this modality has the disadvantage of causing ionizing radiation [10].

Recently, different approaches for CT-like imaging based on magnetic resonance imaging (MRI) have been proposed. Zero echo time (ZTE) imaging has been successfully used to assess the temporomandibular joint (TMJ) and was comparable to CBCT [11]. Hövener et al. compared ultra-short echo-time (UTE) and ZTE imaging of dental pathologies with CBCT and found comparable results due to the ability of UTE and ZTE to detect the fast-decaying signal of solid tissues (e.g., teeth and bone) [12]. In a different study, UTE sequences were used for bone imaging and showed comparable results to CBCT when assessing quantitative and qualitative aspects of medication-related osteonecrosis of the jaw (MRONJ) [13]. To further reduce acquisition times, Getzmann et al. reduced the radial acquisitions of a standard UTE sequence and were able to successfully assess osteolytic lesions and productive bony lesions in patients with MRONJ [14]. In contrast to CBCT, MRI-based UTE and ZTE imaging do not only provide information about osseous structures but also information about soft tissue [12]. As a different approach, Deppe et al. proposed the use of CT-like images based on susceptibility-weighted imaging (SWI) and were able to show comparable results to CT when assessing the sacroiliac joint [15]. More recently, the acquisition of CT-like images based on a three-dimensional (3D) T1-weighted spoiled gradient echo (T1 GRE) sequence has shown promising results when assessing osseous pathologies of the spine and shoulder [16, 17]. Furthermore, CT-like imaging based on a 3D T1 GRE has been used to successfully assess periosteal growth pattern in malignant and benign bone tumors [18].

However, according to the ALARA principle (as low as reasonably achievable), radiation exposure should be reduced to the lowest possible level in order to keep the risk of radiation-associated consequences as low as possible. Consequently, the application of non-radiation-based imaging, such as CT-like MRI, in the setting of acute traumatic mandibular fractures, could have considerable benefit in a young population at risk of radiation accumulation in sensitive regions [10, 19]. Therefore, the purpose of this study was to assess the diagnostic capability of CT-like MRI based on a 3D T1 GRE compared to conventional CT in patients with acute traumatic mandibular fractures.

## Materials and methods

### Ethical statement and patient selection

All procedures were conducted according to the principles expressed in the Declaration of Helsinki. Written patient consent was obtained. The prospective analysis was approved by our institutional review board (Ethics Commission of the Medical Faculty, Technical University of Munich, Germany; Ethics proposal number 432/18).

All patients who were admitted to the emergency department from May 2019 until March 2020 with acute traumatic mandibular fracture received a conventional CT scan of the viscerocranium as part of their routine clinical diagnostic work-up. Following patients were screened for eligibility. Inclusion criteria were a fracture within the area of mandibular and mental foramen. Exclusion criteria included the following: missing consent, positive history for antiresorptive medication or irradiation, mandibular atrophy (residual height < 15 mm), infected fracture, pathological fracture, claustrophobia, and psychomotor agitation. All patients who consented to the study were enrolled and, with the addition of a 3 T MRI for the purposes of this study, which was obtained within 48 h of admission.

### CT imaging

Each subject received a CT scan of the viscerocranium using either a Siemens Somatom Definition AS + scanner or a Philips IQon Spectral CT scanner. Clinical scan parameters were set according to the clinical routine: collimation, 0.625 mm; pixel spacing, 0.3/0.3 mm; pitch factor, 0.6; tube voltage (peak), 120 kVp; modulated tube current, 102–132 mA. Images were acquired in axial orientation and reformatted in sagittal and coronal orientation using a bone-specific convolution kernel (170H/YB, 3 mm slices).

### MR imaging

All subjects were examined using a 3-Tesla MRI scanner (Ingenia; Philips Healthcare, Best, The Netherlands) with dedicated 16-channel head, neck, and spine coils (dStream Head Neck Spine coil, Philips Healthcare, Best, The Netherlands). The following sequences were acquired: (1) 3D short tau inversion recovery (STIR) sequence with the following parameters: echo time, 184 ms; repetition time, 2300 ms; acceleration factor, 2.5; voxel size (acquisition), 0.65 × 0.65 × 1.0 mm^3^; slice number, 180, acquisition time, 6.03 min; (2) 3D double echo steady state (DESS) sequence, echo time, 50 ms; repetition time, 2450 ms; acceleration factor, 2.5; voxel size (acquisition), 0.55 × 0.65 × 1.0 mm^3^; slice number, 360, acquisition time, 5.39 min; and (3) 3D T1 GRE (detailed sequence parameters are displayed in Table 1). The sequences were acquired in axial orientation and reformatted in sagittal and coronal orientation. Additionally, the T1 GRE images were inverted to resemble a bright CT-like bone contrast.

**Table 1:** MRI parameters for the CT-like sequence used in this study

Sequence	3D T1 GRE
Echo time (ms)	1.75
Repetition time (ms)	10
Acceleration factor	2.3
Matrix	420 × 419
Field of view (mm)	180
Voxel size (acquisition, mm^3^)	0.43 × 0.43 × 0.5
Slice number	360
Acquisition time (min)	5:31

### Image analysis

The inverted CT-like MR-based images as well as conventional CT images were analyzed by two radiologists (N.S. and E.B., both radiologists with over 6 years of experience). The images were read individually and independently in random order and blinded to clinical or other diagnostic information. Image analyses were performed on a picture archiving and communication system (PACS) workstation certified for clinical use (IDS7 21.2; Sectra, Linköping, Sweden). The MRI and CT images were read with at least 8 weeks in between readings, respectively. For intra-reader reproducibility, 7 patients were assessed once again after 8 weeks by both radiologists.

### Image analysis and measurements

Imaging was evaluated for the presence and location of mandibular fractures using the findings from conventional CT imaging as a standard of reference. The fractures were classified according to Dingman and Natvig depending on the location of the fracture: symphysis, parasymphysis, body, angle, ramus, condylar process, coronoid process, and alveolar process [20]. Furthermore, the distance of the fracture fragments was measured in all three orientations (axial, coronal, and sagittal) and the mean differences (MDs) were calculated for each orientation as well as in between the modalities. Visibility of fracture lines and the cortical border as well as the bone-to-soft-tissue contrast was evaluated using a 5-point Likert scale (1 = poor, 2 = below average, 3 = fair, 4 = good, 5 = excellent). Overall diagnostic image quality, image artifacts and diagnostic confidence were graded once by both raters also using this five-point Likert scale.

### Statistics

Agreement of ordinal-scaled parameters was assessed using weighted Cohen’s κ [21]. The agreement of numerical data was evaluated with intraclass correlation coefficients (ICCs). The inter- and intra-rater agreement were also calculated using Cohen’s κ and ICCs, respectively [22]. Descriptive statistics were performed using paired *t*-tests (for numeric variables) and McNemar’s tests (for binary categorical variables). All statistical tests were performed two-sided and a level of significance (α) of 0.05 was used. The data were analyzed using IBM SPSS Statistics for Windows (version 27.0; IBM Corp., Armonk, NY, USA).

## Results

**Fig. 1:**
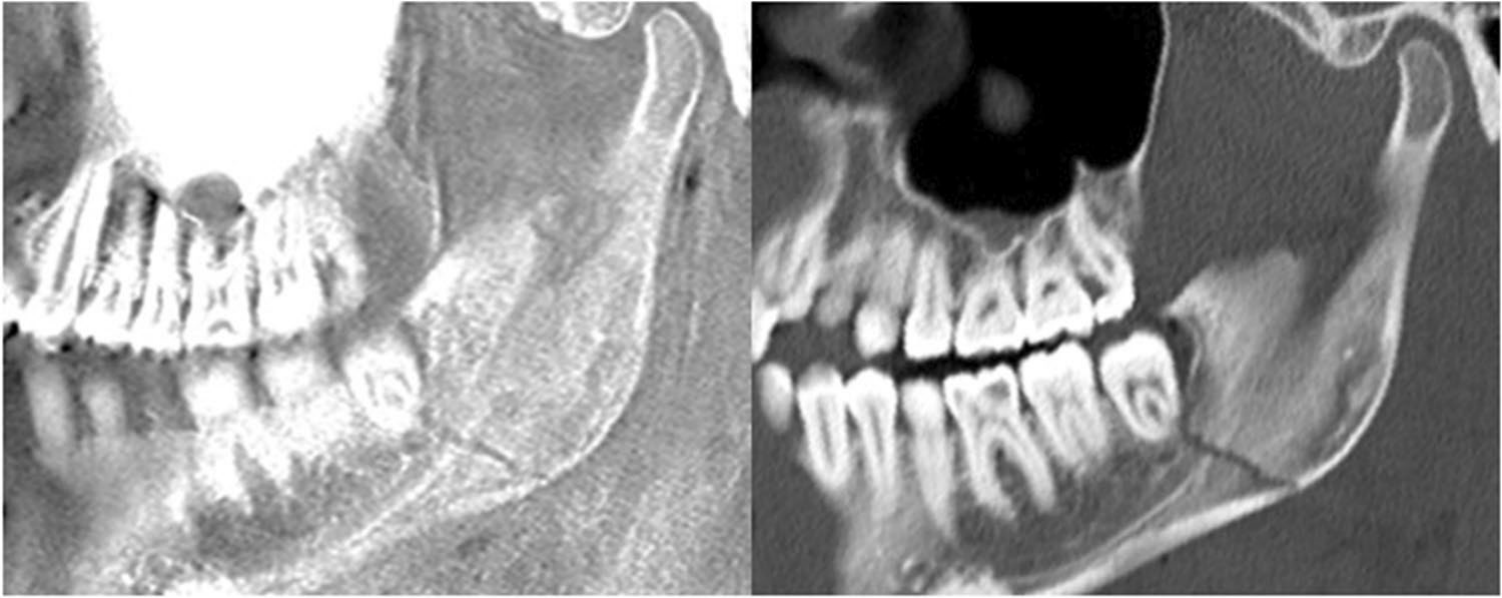
(Left) inverted CT-like MRI based on a 3D T1 GRE sequence in a 23–years-old patient with an acute traumatic mandible fracture after a physical assault. Note, the clearly depicted fracture line compared to the conventional CT (right)

**Fig. 2:**
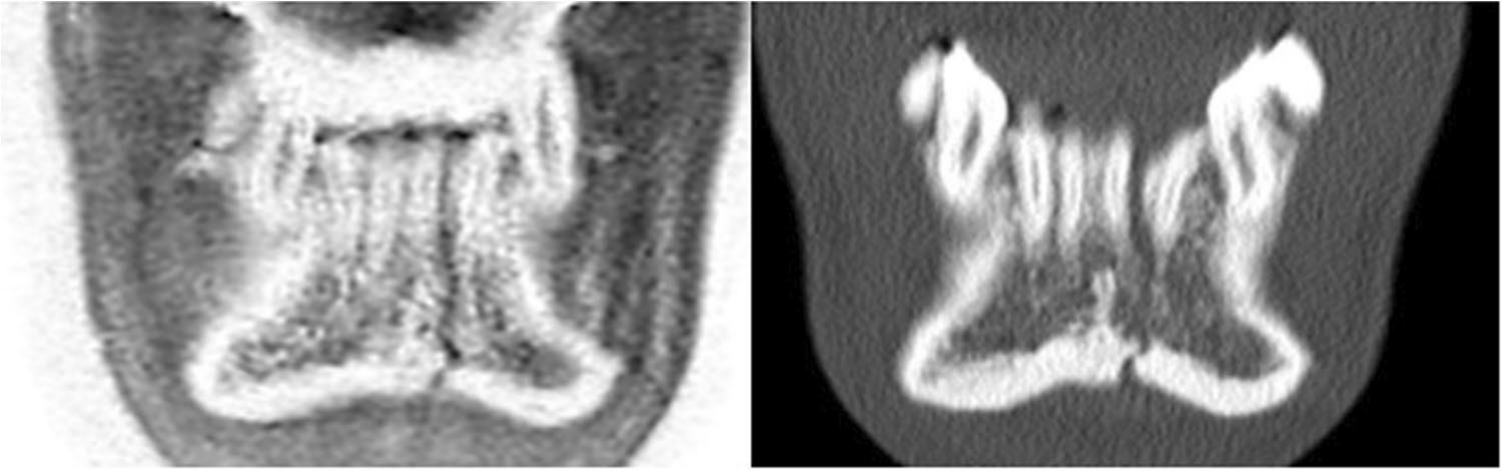
(Left) acute parasymphyseal fracture of the mandible of a 26-years-old patient after a car accident. Note the thin fracture line on the inverted CT-like images compared to the conventional CT (right). An association with the tooth root is assessable on CT-like MRI

**Fig. 3:**
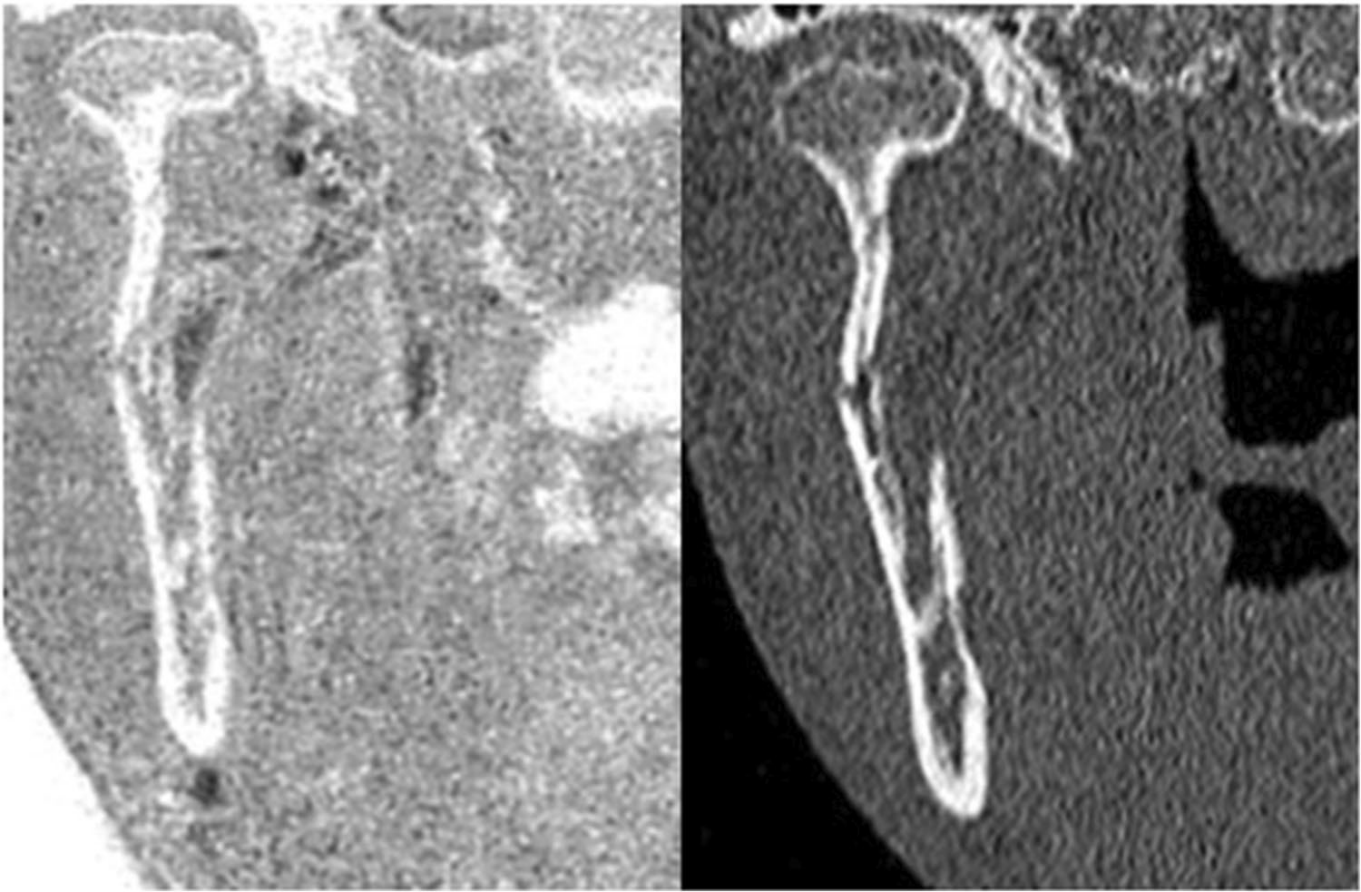
Thirty-two-year-old patient with an acute fracture of the right mandibular ramus after an e-scooter accident (right). Inverted CT-like MRI shows the thin fracture lines and fracture displacement corresponding conventional CT (left)

In total, 14 patients (age 26 ± 3.9 years; 4 females, 10 males) with diagnosed acute mandibular fractures on CT were included into the study. All mandibular fractures were accurately detected using CT-like MRI-based images reconstructed from a 3D T1 GRE by both raters (*n* = 22, κ 1.00 (95% confidence interval (CI) 1.00–1.00), Figs. 1, 2, and 3). Using CT as the standard of reference for fracture detection, mandibular fracture location was classified as symphyseal (*n* = 1), parasymphyseal (*n* = 6), body (*n* = 5), angle (*n* = 7), ramus (*n* = 3), coronoid process (*n* = 0), and alveolar process (*n* = 0).

The overall agreement for the fracture classification between conventional CT and CT-like MRI was excellent (κ 0.93 (95% *CI* 0.90–1.00)). The agreement of fracture classification between reader 1 and reader 2 on the CT-like images was substantial to almost perfect (κ 0.95 (95% *CI* 0.92–1.00)). There was no significant difference between the measurements of the fracture dislocation between conventional CT and CT-like MRI (axial mean difference (MD) 0.06 mm, standard deviation (STDEV) 0.65 mm, *p* = 0.93, coronal MD, 0.08 mm, STDEV 0.59 mm, *p* = 0.89 and sagittal MD, 0.04 mm, STDEV 0.75 mm, *p* = 0.96, Table 2). The overall agreement between the fracture dislocation measurements of readers 1 and 2 on the CT-like MRI data was substantial to almost perfect (*ICC* 0.96 (95% *CI* 0.92–1.00), Table 3).

**Table 2:** Overall mean measurements for degree of fracture dislocations

Parameters			
	CT-like MRI	Conventional CT	*p*-value
Fracture dislocation			
Axial (mm)	2.86 ± 3.1	2.92 ± 3.1	0.93
Coronal (mm)	2.69 ± 2.7	2.77 ± 2.8	0.89
Sagittal (mm)	3.15 ± 3.5	3.15 ± 3.6	0.96

Data are presented as means ± standard deviations

**Table 3:** Agreement between the CT-like MR images and conventional CT

Parameters	T1 GRE based CT-like images and conventional CT	
	Rater 1	Rater 2
Fracture dislocation		
Axial^1^	0.98 [0.96–1.00]	0.97 [0.94–1.00]
Coronal^1^	0.92 [0.90–1.00]	0.98 [0.96–1.00]
Sagittal^1^	0.96 [0.92–1.00]	0.90 [0.89–1.00]
Fracture classification^2^	0.92 [0.90–1.00]	0.93 [0.90–1.00]
Image quality^2^	0.83 [0.58–1.00]	0.89 [0.68–1.00]
Bone to soft tissue contrast^2^	0.91 [0.73–1.00]	0.87 [0.68–1.00]
Artifacts^2^	0.92 [0.72–1.00]	0.83 [0.57–1.00]
Diagnostic confidence^2^	0.88 [0.60–1.00]	0.93 [0.77–1.00]

^1^Interclass correlation coefficient (ICC). ^2^Weighted Cohen’s kappa (κ), data are given with 95% confidence interval

The overall mean image quality of the CT-like MRI data measured on a 5-point Likert scale was good to excellent (mean 4.3 ± 0.6), with substantial agreement between readers 1 and 2 (κ 0.86 (95% *CI* 0.53–1.00)). Image artifacts graded with a 5-point Likert scale were few to absent (mean artifacts 4.45 ± 0.6). No severe artifacts were detected in the CT-like MRI data. Bone-to-soft-tissue contrast of the CT-like T1 GRE images was rated excellent by both readers (overall mean 4.7 ± 0.44), as well as the diagnostic confidence (overall mean 4.8 ± 0.40).

### Intra-rater agreement for fracture dislocation and classification

After at least 8 weeks, both raters reassessed the images of seven randomly chosen patients separately, independently, and blinded to all clinical information. The intra-rater agreement for the measurements of fracture dislocation on CT-like MRI was substantial to almost perfect (*ICC* 0.92 (95% *CI* 0.89–1.00)). All acute mandible fractures were once more accurately identified on the CT-like MRI data (*n* = 22, κ 1.00 (95% *CI* 1.00–1.00) for both raters).

## Discussion

In this study, we were able to demonstrate that the detection and assessment of acute traumatic mandible fractures using CT-like MRI based on a 3D T1 GRE sequence are feasible and accurate compared to conventional CT. All fractures were detected by both raters and the agreement regarding the fracture classification as well as the measurements of fracture dislocation was excellent (κ > 0.9). Both raters reported high CT-like bone-to-soft-tissue contrast as well as a high image quality.

Recently, Lee et al. were able to show that CT-like imaging of the viscerocranium, in particular, the TMJ, was feasible and accurate using ZTE imaging [11]. However, in contrast to our study, mostly healthy participants were assessed with no acute traumatic injuries [11]. In this study, we evaluated acute mandibular fractures using CT-like images based on MRI. Acute mandibular fractures most often occur in young patients, who should be protected from radiation exposure as good as possible [1]. Especially, CT of the viscerocranium causes radiation exposure to the eye lens, which is the most radiation-sensitive organ in the human body [19]. Therefore, CT-like imaging based on MRI might be an alternative to conventional CT, thus completely avoiding any radiation exposure to the patient.

Since several years, evidence on the feasibility and accuracy of CT-like MRI for fracture detection has been increasing. A study by Schwaiger et al. recently showed that the assessments of acute and old vertebral fractures as well as degenerative changes are feasible and accurate using CT-like MRI based on a 3D T1 GRE sequence [16]. Specifically, GRE sequences are widely available nowadays with most scanners and easy to implement in the scan protocol. No additional hardware or specific software is needed to acquire and interpret the images. Furthermore, the geometric accuracy of the deployed 3D T1 GRE sequence compared to CT and CBCT has been shown before [23]. Furthermore, MRI does not only give information about the osseous anatomy but at the same time acquires information regarding trauma to the surrounding soft tissue. Particularly, fractures located at the corpus mandibulae and the lingual part of the angle of the mandible can be associated with direct trauma to the inferior alveolar nerve (IAN) but also less common to the lingual nerve which can lead to neurosensory deficits [24]. A total scan time of approximately 5 min and 30 s seems a reasonable time frame even in routine diagnostics. Further reduction of the scan time using methods such as compressed sensing or deep-learning-based applications should be evaluated in further studies as there have been already some promising approaches [25].

There are certain limitations to our study that need to be addressed. First, the patient cohort was limited in size with mostly young patients that were able to lie motionless for the duration of the MRI examination. In older patients with comorbidities or children, it might be more difficult to maintain a high image quality without movement artifacts. Furthermore, only patients with diagnosed acute mandibular fractures on CT were included into the study without healthy controls. The raters were blinded to all clinical information and the images were read independently and separately but nevertheless this displays a certain bias regarding the patient selection. No patients with metal implants or fractures due to osteolytic bone destruction (e.g., due to metastasis) have been included in this study. Especially in young patients undergoing orthodontic treatment image quality can be considerably impaired. Compared to inverted UTE/ZTE images, ligaments, menisci, and other tissues with short T2 times appear bright on the inverted T1 GRE images and might impair image analysis, e.g., in the knee or spine [26]. As the mandible is mostly surrounded by muscle, this effect did not interfere with the image analysis in this study. However, assessment of the maxilla might be more difficult due to the air-filled maxillary sinuses. The field of view of the T1 GRE sequence was chosen to focus on the mandible and jaw. Partially included regions like the orbital floor were prone to artifacts and not included in this study. This implicates the risk of missing relevant pathologies not included in the field of view.

## Conclusion

Assessment of acute traumatic mandibular fractures might be feasible and accurate using CT-like images based on a 3D T1 GRE sequence and could be comparable to conventional CT. Hence, CT-like MRI might be a useful alternative to conventional CT to reduce radiation exposure.

## Abbreviations

2D: Two-dimensional
3D: Three-dimensional
T1 GRE: T1-weighted spoiled gradient echo
STIR: Short tau inversion recovery
DESS: Double echo steady state
IAN: Inferior alveolar nerve
CBCT: Cone beam computed tomography
CT: Computed tomography
MD: Mean difference
MRI: Magnetic resonance imaging
MRONJ: Medication-related osteonecrosis of the jaw
PACS: Picture archiving and communication system
SWI: Susceptibility-weighted imaging
TMJ: Temporomandibular joint
UTE: Ultrashort echo-time
ZTE: Zero echo time
